# Adaptive Step RRT*-Based Method for Path Planning of Tea-Picking Robotic Arm

**DOI:** 10.3390/s24237759

**Published:** 2024-12-04

**Authors:** Xin Li, Jingwen Yang, Xin Wang, Leiyang Fu, Shaowen Li

**Affiliations:** Key Laboratory of Agricultural Sensors, Ministry of Agriculture and Rural Affairs, School of Information and Artificial Intelligence, Anhui Agricultural University, Hefei 230036, China

**Keywords:** tea picking, robotic arm, path planning, local obstacle avoidance, AS-RRT* algorithm

## Abstract

The Adaptive Step RRT* (AS-RRT*) path planning algorithm for tea-picking robotic arms was proposed as a solution to the autonomy, safety, and efficiency problems inherent to tea-picking robots in tea plantations. The algorithm employs an accumulator-based sampling point selection strategy to enhance the efficiency of path planning and the quality of the resulting path. It combines fast connectivity and pruning optimization methods to identify collision-free paths in a shorter time and to reduce the computational burden. It also incorporates a dynamic step length adjustment mechanism following collision detection, ensuring that the robot arm can avoid obstacles in real time. Furthermore, the generated paths were optimized through the introduction of redundant node removal and curve smoothing techniques. In the robotic arm motion planning experiments, the depth vision sensor was employed to obtain three-dimensional information within the tea plantation as the data source. The experimental results demonstrate that the AS-RRT* algorithm reduces the path length by 14.18% and the path planning time is less than 1 s, indicating that the proposed method enhances the efficiency of path planning and obstacle avoidance performance of the tea-picking robot arm.

## 1. Introduction

China is the primary region for tea production, particularly known for high-quality teas like “single bud”, “one bud with one leaf”, and “one bud with two leaves”, which require precise picking standards. However, the tea plantation environment has distinctive characteristics that make automated picking significantly challenging. Firstly, the branches of tea trees are densely and irregularly distributed, creating a complex three-dimensional obstacle space, which increases the difficulty of obstacle avoidance and path planning for the robotic arm. Additionally, the target tea buds are small and unevenly distributed, placing high demands on the positioning accuracy and flexibility of the robotic arm [1,2,3]. At present, mechanical harvesting methods struggle to achieve the accuracy needed for these premium teas. As a result, tea harvesting mainly relies on manual labor, which is not only inefficient and labor-intensive but also exacerbates the “labor shortage” issue, especially during the spring picking season [4]. Studies show that tea buds have a growth cycle of approximately one week; if they are not picked within this timeframe, the optimal harvest period is missed. Additionally, labor costs constitute roughly 60% of the income of tea farmers [5,6,7].

As robotics and artificial intelligence technologies advance, intelligent tea bud-picking systems are emerging as an important area of research and application [8,9,10,11]. Traditional path planning methods struggle to meet the real-time and accuracy requirements in the tea plantation environment. The complex spatial distribution and dense obstacles within the plantation further increase the difficulty and computational complexity of path planning. In recent years, both domestic and international researchers have made significant progress in addressing the challenge of motion planning for robotic arms in high-dimensional spaces and with multiple degrees of freedom [12,13,14,15]. The main approaches to path planning fall into two categories: graph search-based methods, such as Dijkstra’s and A* algorithms [16], and sampling-based methods, including the Probabilistic Roadmap Method (PRM) and Rapidly exploring Random Trees (RRT) [17,18]. RRT has become particularly popular in robotic arm path planning due to its capability to effectively search high-dimensional spaces. However, despite its advantages, the RRT algorithm often faces challenges with low sampling efficiency and suboptimal path quality [19,20,21,22].

To address the limitations of standard RRT algorithms [23,24,25,26,27,28,29,30,31,32,33,34]—such as low sampling efficiency and suboptimal path quality—researchers have developed various enhancements focused on reducing sampling randomness [26,27], increasing search speed [27,28], and improving path cost and quality [30,31]. For example, the target gravity RRT algorithm proposed in the literature [32] integrates concepts from the artificial potential field method, introducing a target “gravity” effect in the sampling process that enhances bias toward the target, thereby improving both search efficiency and convergence speed. Similarly, the RRT Connect algorithm [33] accelerates convergence by using bidirectional growth, where two trees extend from the start and target points, respectively, meeting in the middle to form a complete path.

Other improvements include the RRT* algorithm [34], which incorporates random geometric graphs and pruning mechanisms, ensuring asymptotic optimality and minimizing path complexity. The Quick RRT* algorithm [35] further optimizes RRT* by applying the principle of triangular inequality to refine the process of parent node reselection and rewiring, yielding better initial path quality [36,37]. Although the RRT* algorithm progressively optimizes generated paths and reduces path costs, it still suffers from high memory consumption and lengthy computation times when dealing with a large number of nodes. These limitations hinder its application in tasks requiring high real-time performance. For tea plantation picking robots, the challenges posed by complex 3D obstacle environments and stringent accuracy requirements further exacerbate these issues, making it difficult for the algorithm to meet the demands of real-time path planning effectively.

In the realm of applied research on picking robots, various path planning algorithms have been explored to address the challenges posed by complex environments [38,39,40,41,42,43]. For instance, Li Xian et al. [44] developed a path planning system for tea-picking robots using the RRT algorithm. However, its efficiency remains constrained when navigating through dense obstacles. To enhance sampling efficiency and search speed, Zhang Qin et al. [45] introduced the CTB-RRT* algorithm, which incorporates techniques like Cauchy sampling and dynamic adjustments to the tree growth direction. Despite these advancements, further improvements in path optimization and smoothness are still necessary. Additionally, Liu Dun et al. [46] proposed an Informed-RRT*-based path planning method for a citrus-picking robotic arm, aiming to address the time-consuming and inefficient nature of existing robotic arm path planning solutions in agricultural settings.

Therefore, building upon the improvement concepts of the RRT algorithm, this paper proposes an Adaptive Step Size RRT (AS-RRT*) algorithm to address issues of sampling randomness, inadequate path planning quality, and the challenges associated with collision detection adjustments. By implementing an accumulator-based sampling point selection strategy, integrating rapid connectivity and pruning optimization methods, adopting a dynamic step length adjustment mechanism, and introducing techniques for redundant node removal and curve smoothing, the algorithm aims to enhance the path planning efficiency and safety of tea-picking robots in tea plantation environments. These improvements seek to reduce path lengths and alleviate computational burdens.

## 2. Tea Plantation Environment and Path Planning Methods

### 2.1. Characteristics of Tea Plantation Environment for Picking

The tea plantation environment features complex and varied terrain, including irregular tea tree heights, branch and leaf densities, and tea bud locations, all of which complicate path planning. Key challenges include unstructured space, where the irregular distribution of obstacles and dynamic changes in the tea tree growth environment due to seasonal shifts or agricultural activities increase the difficulty of obstacle avoidance; high-density obstacles, with tea tree branches and leaves closely packed and tea buds densely distributed, requiring the robotic arm to navigate narrow spaces without damaging the tea tree or buds; and real-time requirements, where the path planning algorithm must generate stable and feasible paths within limited time constraints to meet the speed and safety needs of real-time operations.

These factors together present the path planning problem, where the key is to balance picking efficiency with operational safety under complex conditions, ensuring the robotic arm’s end-effector accurately reaches the target picking point.

### 2.2. Robotic Arm Kinematics Modeling

**Figure 1:**
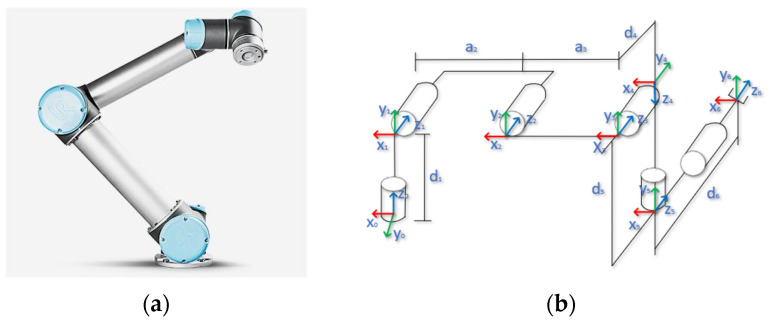
UR5 robotic arm. (**a**) Robotic arm physical model; (**b**) robotic arm D-H model.

To meet the demands of the tea plantation environment, the UR5 robotic arm manufactured by Universal Robots in Denmark was selected as the hardware platform for the picking system in this study, as shown in Figure 1a. The UR5 robotic arm, with six rotational degrees of freedom, possesses high flexibility and can meet the requirements of complex tea plantation picking tasks. To accurately control the motion trajectory of the end-effector, the Denavit–Hartenberg (D-H) parametric method was employed to establish the kinematic model of the UR5 robotic arm (Figure 1b). The model parameters are listed in Table 1.

The Denavit–Hartenberg (D-H) parameters are defined as follows: $\alpha_{i-1}$ denotes the linkage torsion angle, which describes the rotational relationship between neighboring linkages. $a_{i-1}$ represents the linkage length, which is the distance between two neighboring joints along the *x*-axis. $\theta_{i-1}$ is the joint angle, which is the control input variable that defines the rotation of the joint. $d_{i}$ is the linkage offset, which is the distance between two neighboring joints along the *z*-axis. Using these parameters, the transformation matrix that describes the relationship between the i-th joint and the (I − 1)-th joint can be expressed as follows:

(1) $$T_{i}=\left[\begin{array}{cccc} {cos\theta }_{i} & {-sin\theta }_{i}{cos\alpha }_{i} & {{sin\theta }_{i}sin\alpha }_{i} & {a_{i}cos\theta }_{i}\\ {sin\theta }_{i} & {cos\theta }_{i}{cos\alpha }_{i} & {-cos\theta }_{i}{sin\alpha }_{i} & {a_{i}sin\theta }_{i}\\ 0 & {sin\alpha }_{i} & {cos\alpha }_{i} & d_{i}\\ 0 & 0 & 0 & 1 \end{array}\right]$$

This matrix describes the position and orientation of the i-th joint relative to the (I − 1)-th joint, with each term corresponding to the D-H parameters defined above. By sequentially multiplying the transformation matrices of all the joints, the transformation of the robotic arm’s end-effector relative to the base coordinate system can be obtained, thereby establishing the kinematic equations of the robot.

(2) Tn0=T⋯10T⋯ii−1Tnn−1

To enhance the reliability and safety of path planning, collision detection is optimized in this study. Each linkage of the UR5 robotic arm is approximated as a cylinder, while irregular obstacles in the tea plantation are modeled by surrounding them with spheres or rectangular bodies. This approach simplifies the collision detection task to an intersection problem between regular geometries, thus improving computational efficiency and ensuring the feasibility and safety of path planning in the complex tea plantation environment.

### 2.3. RRT* Algorithm

The RRT* algorithm enhances the original RRT algorithm by optimizing the selection of parent nodes. Its fundamental principle involves selecting the node with the minimum path cost as the parent node and subsequently reconnecting all nodes to compute an asymptotically optimal solution. The algorithm comprises two main steps: parent node selection and random tree reconnection [47].

In the process of reselecting the parent node for the new node $x_{new}$, a set of candidate nodes $x_{nearSet}$ is generated by searching for nearby nodes within a specified search radius centered on $x_{new}$. Next, the Euclidean distances from each candidate node in the set to the starting point are computed individually and combined with the Euclidean distance from $x_{new}$ to each candidate node to obtain the total distance. By comparing these total distances, the candidate node $x_{min}$ with the smallest distance is identified and selected as the parent node of $x_{new}$, facilitating the reselection of the parent node.

**Figure 2:**
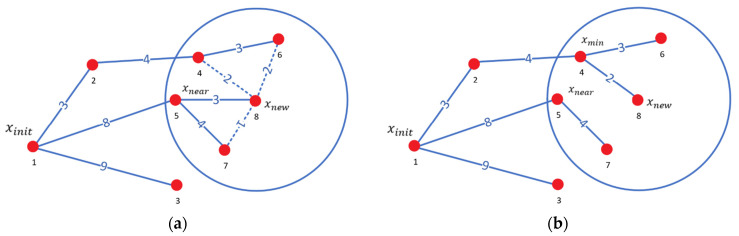
RRT* algorithm reselects parent node process. (**a**) Reselecting the parent node process; (**b**) reselect parent node result. The red dots indicate path nodes.

As illustrated in Figure 2 and Figure 2a, node 8 represents the newly generated node $x_{new}$. The node labels indicate the order of generation, while the numbers between two nodes denote the Euclidean distances between them. When reselecting the parent node, a search radius is defined with node 8 as the center of the circle, identifying nearby nodes 4, 6, and 7 as candidate nodes within this range. The original path 1-5-8 has a cost of 11, while the alternative paths are as follows: 1-2-4-8 with a cost of 9, 1-2-4-6-8 with a cost of 12, and 1-5-7-8 with a cost of 13. Consequently, node 4, which exhibits the lowest path cost, is selected as the parent node for node 8. Figure 2b displays the updated path after the parent node reselection.

After reselecting the parent node for $x_{new}$, the RRT* algorithm rewires the random tree. This process begins by excluding the closest parent node $x_{min}$ from the re-evaluation of candidate nodes. The Euclidean distance between each remaining candidate node and $x_{new}$ is then calculated and added to the cost of the path from $x_{new}$ back to the starting point $x_{init}$. If the total cost of this new path is less than the original path cost for a given candidate node, the parent node of that candidate node is updated to $x_{new}$, thereby rewiring the path.

**Figure 3:**
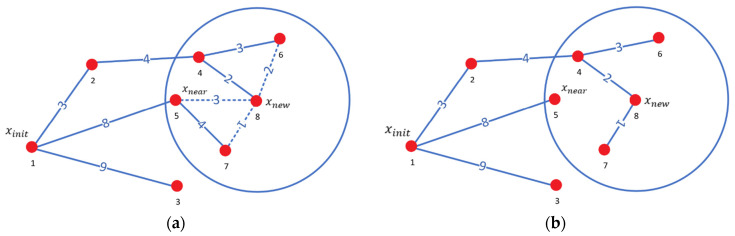
RRT* algorithm rewires the random tree process. (**a**) Rewire the random tree process; (**b**) rewiring the random tree result. The red dots indicate path nodes.

As illustrated in Figure 3, in Figure 3a, node 8 represents the newly generated node $x_{new}$, with nearby nodes including nodes 5, 6, and 7. Their respective parent nodes are 1, 4, and 5, with path costs of 8, 10, and 12, respectively. If the parent node of node 5 were changed to $x_{new}$, the path cost would increase to 12, which is greater than the original cost of 8. Therefore, the parent node of node 5 remains unchanged. Similarly, the parent node of node 6 remains unchanged. However, for node 7, changing its parent node to $x_{new}$ reduces its path cost from 12 to 10, so node 8 is selected as the new parent node of node 7. Figure 3b displays the updated path after rewiring. Each step of rewiring presents an opportunity to further reduce the overall path cost, thereby optimizing the path of the entire random tree.

## 3. AS-RRT* Algorithm

### 3.1. Accumulator-Based Sampling Point Selection Strategy

In the tea plantation environment, the branches and leaves of tea trees are densely and irregularly distributed. While the RRT* algorithm extends nodes using random uniform sampling, which provides strong exploratory capabilities, the randomness in its search direction often results in a large number of invalid nodes in this complex environment, significantly reducing search efficiency. Additionally, biased sampling or target point expansion strategies can sometimes lead the algorithm into local minima, thereby impacting its performance.

To address these problems, a dynamic step size adjustment strategy based on an accumulator (ACC) is proposed to enhance sampling efficiency, specifically tailored for the unstructured space and high-density obstacles characteristic of tea plantations. This approach dynamically adjusts the step length using the ACC to balance exploration and precise searching. During the random tree expansion process, the algorithm initially applies a random sampling strategy. When a sampling point is successfully expanded (i.e., no obstacles are encountered), the ACC is incremented; if expansion fails (i.e., obstacles are encountered), the ACC is reset to zero, as expressed in Equation (3).

(3) $$ACC\left(t\right)=\left\{\begin{array}{c} \mathrm{ACC}\left(t-1\right)+1\;(\mathrm{if}\;\mathrm{sample}\;\mathrm{expanded}\;\mathrm{successfully})\\ 0\;(\mathrm{if}\;\mathrm{sample}\;\mathrm{expanded}\;\mathrm{failed}) \end{array}\right.$$

where ACC(t) represents the value of the accumulator during the current sampling round t, reflecting the current sampling state. The step size is dynamically adjusted based on the accumulation of ACC, as expressed in Equation (4):

(4) $${step}_{c}=\left\{\begin{array}{c} {step}_{init}+a\times ACC\;(ACC\leq \theta )\\ \mathrm{Max}({step}_{init},{step}_{init}-b\times \mathrm{ACC})\;(\mathrm{ACC}>\theta ) \end{array}\right.$$

In this equation, ${step}_{c}$ represents the current step size while ${step}_{init}$ denotes the initial step size. When the ACC value increases, it indicates that the current region has a lower density of obstacles, prompting the step size to increase gradually and thereby accelerating the path search process. However, once ACC exceeds the predefined threshold, the step size is gradually reduced to enhance the accuracy of the path search and prevent potential paths from being overlooked. Simultaneously, the step size is not excessively reduced but maintained at least at the initial value to ensure a reasonable search speed. Additionally, the direction of sampling point generation is optimized to bias it toward the target point, facilitating a faster convergence toward the goal. The range of sample point generation is determined using the following equation:

(5) $${range}_{t}{={point}_{t}+{step}_{c}\times direct}_{v}$$

**Figure 4:**
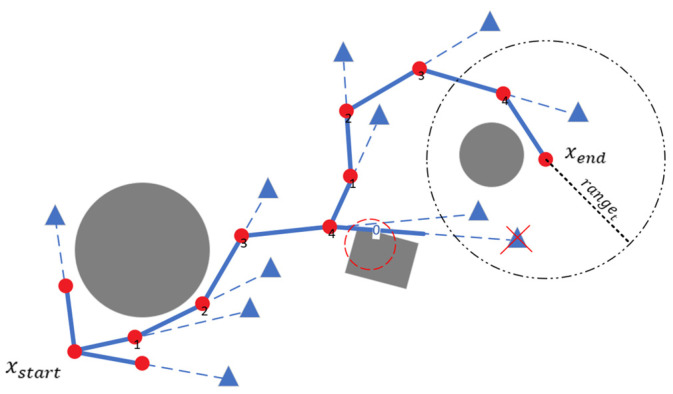
Random point sampling process. The red dots indicate path nodes, triangles indicate random sampling points, red circles denote collisions, and ‘X’ marks indicate discarded sampling points.

The direction vectors are calculated as follows:

(6) $${direct}_{v}={direct}_{t}\times {ACC}_{n}+{direct}_{r}\times (1-{ACC}_{n})$$

where ${range}_{t}$ represents the range of the sampling point, ${direct}_{v}$ denotes the direction of the sampling point, ${direct}_{t}$ indicates the direction of the target point, and ${direct}_{r}$ refers to the random direction. ${ACC}_{n}$ denotes the normalized value of the current accumulator (ACC). As the ACC increases, the value of ${ACC}_{n}$ gradually rises, meaning that the sampling point becomes more inclined toward the direction of the target point. Figure 4 illustrates the random point sampling process, with the ACC threshold set to 3. The figure shows the value of ACC after the completion of a sampling round.

### 3.2. Fast Connectivity and Pruning Optimization Methods

In the RRT* algorithm, the path optimization process, including parent node reselection and pruning, typically involves traversing all the nodes in the current tree and examining the neighborhood of each node to identify potentially optimal parent nodes and prune redundant paths. As each operation requires scanning through the neighborhood nodes, the time complexity of expanding a single new node is O(n), where n is the total number of nodes in the current tree. Consequently, the overall time complexity of the algorithm becomes O(n^2^), significantly increasing the computational burden, particularly when the number of nodes is large.

In the tea plantation environment, the unstructured distribution of obstacles and the irregular positions of tea buds exacerbate the computational effort required for neighborhood traversal as the neighborhood range expands, thereby reducing the efficiency of the algorithm. To address this issue, an optimization method is proposed to minimize computational effort and enhance algorithm efficiency. The pseudo-code for this method is presented in Algorithm 1.
**Algorithm 1:** Fast connectivity and pruning optimization method pseudo-code  1:V ← {start_node}; E ← ∅; T ← (V, E);  2:for ind = 0 to N do  3:new_node ← sample(ind);  4:if collision_free(new_node, start_node) then  // Determine whether $x_{\mathrm{new}}$ and $x_{\mathrm{start}}$ are in collision  5:min_node ← start_node;  //If no collision, connect the new node $x_{\mathrm{new}}$ to the start point $x_{\mathrm{start}}$  6:else  7:// If there is a collision, proceed according to the RRT* process  8:end if  9:end for10:return T = (V, E);

The core of this optimization lies in reducing the algorithm’s time complexity by minimizing neighborhood traversal and pruning computations. After generating a new random point $x_{new}$, the algorithm attempts to connect it directly to the start point $x_{new}$ as an initial step. This direct connection approach is particularly effective in regions with sparse obstacles or fewer tea buds, enabling quick identification of a collision-free path and reducing the need for extensive neighborhood traversal and path cost calculations. This improvement reduces the algorithm’s time complexity to O(n) in environments with fewer obstacles, significantly enhancing its efficiency in less complex scenarios.

During the pruning optimization process, the algorithm prioritizes direct connections to bypass unnecessary neighborhood node traversal and path cost recalculation. If a direct connection is successful, a portion of the pruning operations can be skipped, avoiding redundant computations. This approach further reduces the overall computational workload. This method significantly enhances the efficiency of path planning, particularly in the tea plantation environment where obstacles are irregularly distributed, and the positions of tea buds are non-fixed. By reducing unnecessary calculations, the optimization makes the algorithm more practical and efficient, better suited for real-world applications in complex environments.

### 3.3. Dynamic Step Length Adjustment Mechanism After Collision Detection

Considering the dense distribution of branches and leaves, as well as the requirement for efficient and flexible obstacle avoidance in tea gardens, collision detection is a critical aspect of the RRT* algorithm. Ensuring the stability and safety of the robotic arm during path planning is paramount. However, the intricate arrangement of obstacles and the high density of tea tree branches and leaves in the tea plantation environment greatly increases the complexity of collision detection. To improve the algorithm’s adaptability and enhance the efficiency and robustness of path planning, a post-collision detection adjustment mechanism is introduced. This mechanism dynamically refines path adjustments after detecting collisions, ensuring safe and efficient navigation through the complex and obstacle-dense tea plantation environment.

Specifically, the post-collision detection adjustment mechanism adapts the search strategy by dynamically adjusting the step size, enabling more precise path detection when collisions occur. This is particularly crucial for navigating complex obstacles, such as tea tree branches and leaves, within the tea plantation environment. When a collision is detected, the mechanism reduces the step size, allowing the algorithm to generate sampling points that more accurately avoid obstacles, particularly in the narrow spaces between tea trees. By progressively decreasing the step size and making multiple attempts to generate new sample points, the algorithm is able to explore the intricate interior of the tea plantation, ensuring both the reliability and accuracy of the path planning process.

**Figure 5:**
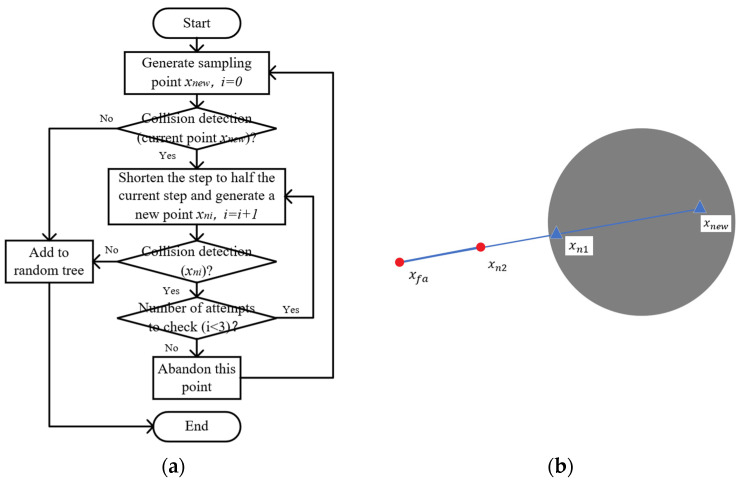
Diagram of dynamic step length adjustment mechanism after collision detection. (**a**) Process diagram; (**b**) schematic diagram.

The implementation process of the dynamic step size adjustment mechanism is outlined as follows, with details provided in Figure 5a. First, collision detection is conducted on the current sampling point. If the detection is successful, the point is added to the random tree. If the detection fails, the algorithm reduces the current step length ${step}_{c}$ by half, generates a new sample point $x_{n1}$, and performs collision detection again. Figure 5b illustrates the mechanism: if collision detection for the new sample point $x_{n1}$ is successful, the path tree is updated, and this point is designated as the new path point. If collision detection fails again and the number of attempts does not exceed three, the algorithm returns to the previous step to continue shortening the step size and generating a new point. After more than three unsuccessful attempts, the algorithm ceases further attempts and proceeds with the remaining process.

### 3.4. Redundant Node Removal and Curve Smoothing

In path planning, the paths generated by the RRT* algorithm are typically formed by connecting nodes in a random tree, which may result in jagged trajectories rather than smooth curves. This can adversely affect the stability of the robot and contribute to increased wear and tear on its components. To mitigate these issues, it is essential to smooth the planned paths.

**Figure 6:**
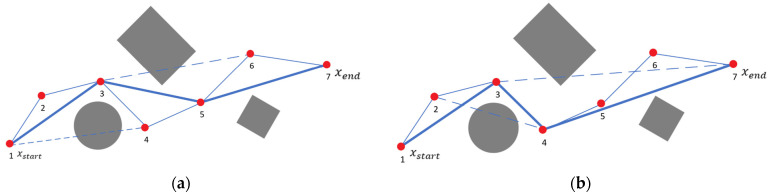
Schematic diagram of redundant node removal. (**a**) From start point to target point; (**b**) from target point to start point. The red dots represent path nodes, and the dashed lines indicate discarded connections due to collisions.

A redundant node removal operation is performed on the generated paths. After obtaining a collision-free set of path points, the algorithm begins connecting these points sequentially until a collision occurs. Upon detecting a collision, the path point immediately before the collision is retained, and this node is designated as a new starting point to re-execute the path connection process until the goal point is reached. Once the path connection is complete, the lengths of the paths—from the starting point to the goal point and from the goal point back to the starting point—are compared, and the shorter path is retained as the final collision-free path. The simplified process is illustrated in Figure 6, where Figure 6a shows the path from the initial point to the goal point, and Figure 6b depicts the path from the goal point back to the initial point. The thin solid line represents the original path, the dashed line indicates the path where the connection attempt failed, and the thick solid line denotes the final collision-free path generated.

**Figure 7:**
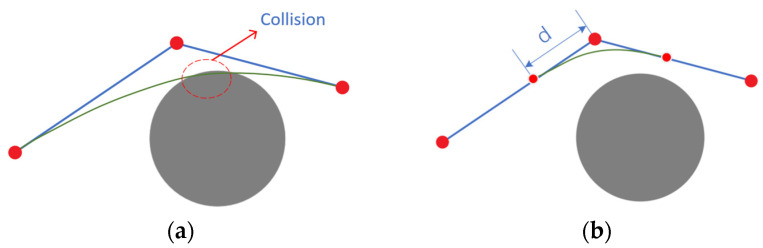
Schematic diagram of local smoothing. (**a**) Cubic B-spline smoothing; (**b**) add control points for smoothing. The blue lines represent the original path, and the green lines represent the smoothed path.

To ensure the simplified path maintains good smoothness, the cubic B-spline curve is introduced to handle the turning points along the path. The cubic B-spline curve represents the path as a weighted sum of control points and associated basis functions. The general equation of the cubic B-spline curve is as follows:

(7) $$P\left(t\right)=\sum_{i=0}^{n}P_{i}F_{i,k}\left(t\right)$$

where $P_{i}$ is the characteristic point of the control curve, and $F_{i,k}\left(t\right)$ is the k-th order B-spline basis function. Directly smoothing the paths after removing redundant nodes may lead to path collisions with obstacles (as shown in Figure 7a). To prevent this, additional path points are inserted near each turning point before the smoothing process. These points are placed both before and after the turning points, with a distance d between them (as shown in Figure 7b). By dynamically adjusting the value of d, the path can be adaptively adjusted based on the length and complexity of the turns: for longer or smoother segments, a larger d is used to enhance smoothing efficiency; for shorter or more frequent turns, a smaller d is selected to keep the path close to the original trajectory and reduce the risk of collision. This setup effectively minimizes the likelihood of path deviation and ensures that the smoothed path closely follows the original trajectory.

In addition, to further optimize path smoothness and reduce the risk of collisions, the distribution of control points is adaptively designed. Specifically, the distance information of each path segment is obtained by calculating the Euclidean distance between neighboring path points:

(8) $${distance}_{i}=\left\|p_{i+1}-p_{i}\right\|,for\;i=1,2,\ldots ,n-1$$

where $p_{i}$ and $p_{i+1}$ denote the positions of two neighboring points in the path, respectively. The distances of these path segments are then summed to obtain the cumulative distance of the path:

(9) $$t_{smooth}=\sum_{i=1}^{n-1}{distance}_{i}$$

To normalize the path smoothing process, the cumulative distance $t_{smooth}$ is normalized as follows:

(10) $$t_{smooth}=\frac{t_{smooth}}{t_{smooth}\left[-1\right]}$$

The normalized cumulative distance $t_{smooth}$ serves as the basis for the distribution of control points. In areas with dense or hazardous obstacles, the smoothness of the path is restricted by increasing the number and density of control points, ensuring effective obstacle avoidance. In contrast, in regions further from obstacles, the density of control points is reduced, allowing for more natural and efficient path smoothing.

## 4. Experiments and Results

### 4.1. Experiment and Analysis of Sampling Point Selection Strategies

To evaluate the performance of the improved AS-RRT* algorithm, experiments were conducted in a Python 3.8 environment. The experimental setup was based on a Windows 10 operating system, with hardware specifications including an Intel(R) Core(TM) i7-9750H CPU running at 2.60 GHz and 16 GB of RAM. Two distinct 3D simulation environments were designed for the experiments, with a map size of 100 mm × 100 mm × 100 mm. The start point was set at (1, 1, 1) and the end point at (100, 100, 100), with obstacles represented as spheres.

The specific settings for the experiments are as follows: Test Environment I features 8 uniformly distributed obstacles, where the space is divided into a uniform grid, and obstacles with a radius of 15 mm are placed at the center of each grid cell. Test Environment II includes 30 randomly generated obstacles, with their locations and radii chosen randomly to better simulate a tea plantation environment. During the experiments, the target bias probability is set to 0.2, the path search step size is 5 mm, and the maximum number of iterations is set to 2000. Considering the inherent randomness of the extended random tree algorithm, each group of experiments was repeated 100 times, and the final results were averaged to ensure reliability and consistency.

#### 4.1.1. ACC Threshold Selection

**Figure 8:**
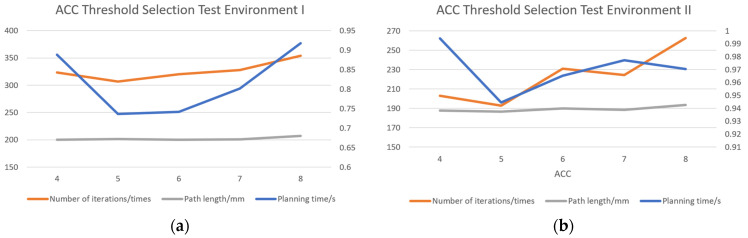
ACC threshold selection. (**a**) Test Environment I; (**b**) Test Environment II.

The accumulator (ACC) is introduced in the sampling point selection strategy, and setting an appropriate ACC threshold is crucial for improving the performance of the algorithm. Experiments are conducted to compare and analyze the planning time, iteration number, and path length by adjusting the ACC threshold. The experimental results are presented in line graphs (Figure 8), clearly illustrating the trends of each index under different ACC thresholds.

In Test Environment I, the shortest planning time (0.74 s), the fewest number of iterations (306.49), and a reasonable path length (201.13 mm) were achieved with an ACC threshold of 5. In Test Environment II, an ACC threshold of 5 also yielded the best results, with a planning time of 0.94 s, 192.84 iterations, and the shortest path length (186.45 mm). The line graphs clearly demonstrate the superior overall performance at an ACC threshold of 5. Therefore, an ACC threshold of 5 is selected as the optimal value in this experiment. This threshold not only accelerates the path search process but also enhances the efficiency and accuracy of the algorithm, particularly in complex environments such as dense, obstacle-rich, and unstructured tea plantation environments.

#### 4.1.2. Stability Analysis of Dynamic Step Adjustment

In order to demonstrate the stability of the sampling point condition selection strategy and the adjustment of the dynamic step size, the experiments were repeated 100 times in different environments. The mean and variance of the step size were calculated, and the results are shown in Table 2.

These data show that in Test Environment I, the mean step size is slightly higher, and the variance is slightly larger, but the fluctuation remains within acceptable limits. The AS-RRT* algorithm was able to maintain relatively stable step size adjustments with an even distribution of obstacles. In contrast, in Test Environment II, the mean step size is slightly lower, and the variance is reduced. This indicates that in more complex obstacle environments, the AS-RRT* algorithm is able to appropriately adjust the step size to respond to environmental changes while maintaining stable step size adjustments.

#### 4.1.3. Convergence Analysis of Dynamic Step Size Adjustment

**Figure 9:**
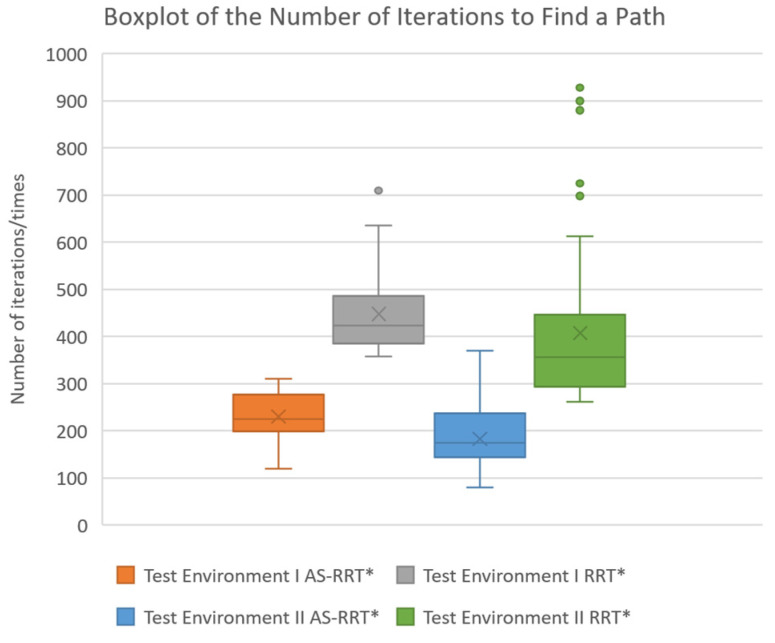
Boxplot of the number of iterations to find a path.

The number of iterations was analyzed using box-and-whisker plots (Figure 9) to provide a more intuitive visualization of the convergence of the sampling point selection strategy under different sampling point conditions in various experimental environments.

In Test Environment I and Test Environment II, the box-and-whisker plots demonstrate that the AS-RRT* algorithm significantly reduces the number of iterations compared to the RRT* algorithm, with a more concentrated distribution. This suggests that the improved algorithm not only reduces the number of iterations during the path search process but also exhibits more stable convergence. Specifically, the narrower spacing between the upper and lower quartiles in the AS-RRT* boxplot indicates a more compact data distribution with no outliers, reflecting the algorithm’s stable and reliable convergence in complex obstacle environments. In contrast, the RRT* algorithm’s boxplot shows a broader distribution range and potential outliers, indicating less stable convergence compared to the AS-RRT* algorithm.

### 4.2. Target Sampling Experiment and Analysis

Based on the optimal ACC threshold of 5 determined in the previous experiments, target sampling experiments were conducted to verify the overall performance of the AS-RRT* algorithm in different experimental environments. With other parameter conditions kept constant, the experiment was repeated 100 times. The results are shown in Table 3 and Table 4, respectively.

**Figure 10:**
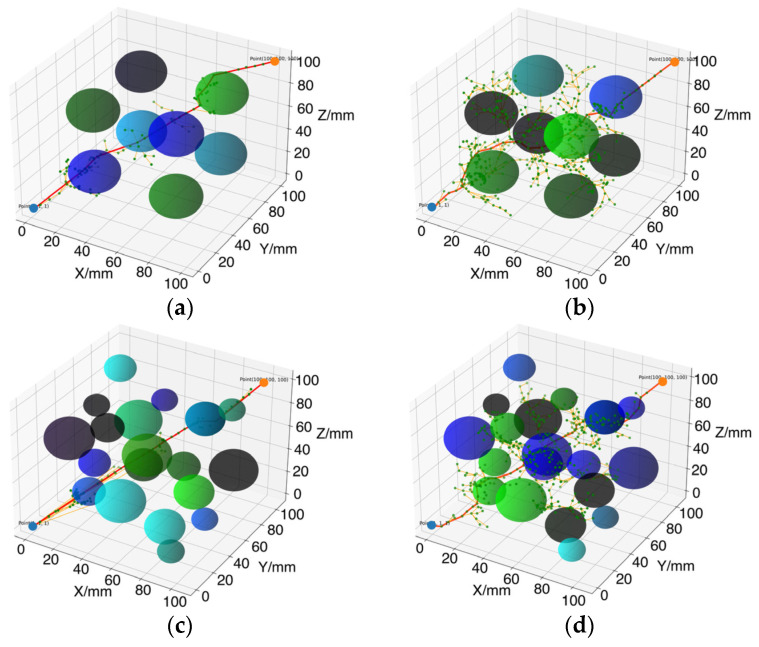
Three-dimensional environmental path planning results map: (**a**) Test Environment I: AS-RRT*; (**b**) Test Environment I: RRT*; (**c**) Test Environment II: AS-RRT*; (**d**) Test Environment II: RRT*. The blue point represents the starting point (1, 1, 1), the yellow point represents the endpoint (100, 100, 100), green points are random sampling points, spheres represent obstacles, the yellow curve shows the randomly expanded path, and the red curve depicts the final path.

The path planning results are illustrated in Figure 10, with the blue point as the starting point, the orange point as the target point, the green points as random sampling points, the spheres representing obstacles, the yellow curve indicating the randomly expanded path starting from the initial point, and the red curve depicting the final generated path. Comparing the path planning diagrams, it can be observed that the AS-RRT* algorithm significantly reduces the usage of random sampling points and the number of randomly expanded paths during the spatial search, resulting in fewer path nodes compared to those generated by RRT*. This indicates that the AS-RRT* algorithm has better guidance and purposefulness during the search process, effectively reducing invalid nodes and sampling points, lowering the overall cost of path planning, and improving search efficiency.

The data presented in Table 3 and Table 4 demonstrate that the AS-RRT* algorithm significantly enhances path planning speed in terms of search efficiency. In Test Environment I, the average planning time for the RRT* algorithm is 0.93 s, while the AS-RRT* algorithm reduces this to 0.51 s, representing a reduction of 45.16%. In Test Environment II, the planning time for the RRT* algorithm is 1.25 s, which the AS-RRT* algorithm further reduces to 0.55 s, achieving a time savings of 56.00%. These results indicate that the AS-RRT* algorithm is more directed in its path search process, effectively decreasing the number of invalid nodes and sampling points, thereby significantly improving search efficiency.

Additionally, the comparison of the number of nodes and sampled nodes further reinforces this conclusion. In Test Environment I, the average number of nodes for the RRT* algorithm is 29.90, with 328.61 sampled nodes. In contrast, the AS-RRT* algorithm reduces the average number of nodes to 19.61 and the number of sampled nodes to 278.02. This indicates that the AS-RRT* algorithm effectively minimizes redundant nodes generated during the path planning process, thereby reducing computational burden. In Test Environment II, the RRT* algorithm yields 31.17 nodes and 257.07 sampled nodes, whereas the AS-RRT* algorithm decreases these figures to 15.23 nodes and 171.37 sampled nodes. This demonstrates that the AS-RRT* algorithm is more targeted in its path search process, enhancing overall efficiency.

In terms of path length, the AS-RRT* algorithm demonstrates notable improvements in the quality of the generated paths. In Test Environment I, the RRT* algorithm achieves a path length of 206.29 mm, while the AS-RRT* algorithm successfully reduces this to 193.00 mm, representing a relative reduction of 6.44%. In Test Environment II, the path length of the RRT* algorithm is 203.50 mm, and the AS-RRT* algorithm further decreases it to 187.77 mm, achieving a relative reduction of 7.73%. These results indicate that the AS-RRT* algorithm effectively generates shorter paths, thereby enhancing the efficiency of the robot’s movement in real-world applications.

Regarding the proportion of validly sampled nodes, the AS-RRT* algorithm exhibits a lower percentage compared to the RRT* algorithm, reflecting its more targeted path search strategy. In Test Environment I, the RRT* algorithm has approximately 65.7% validly sampled nodes, while the AS-RRT* algorithm shows around 57.3%. In Test Environment II, the RRT* algorithm’s proportion is about 73.3%, with the AS-RRT* algorithm decreasing to approximately 64.5%. Despite this slight reduction in the percentage of effectively sampled nodes, it does not indicate a decline in performance. Instead, the AS-RRT* algorithm demonstrates greater adaptability and effectiveness in specific environments by optimizing the path search process. It efficiently selects critical path points while reducing the generation of invalid nodes, ultimately enhancing overall search efficiency and making the AS-RRT* algorithm more effective in generating viable paths.

### 4.3. Path Smoothing Experiment and Analysis

**Figure 11:**
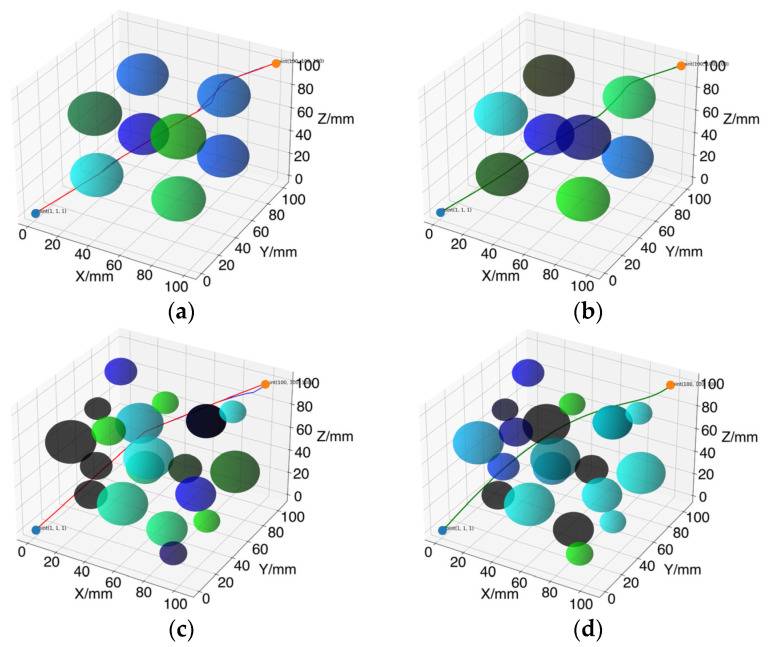
Three-dimensional environmental path optimization results map: (**a**) Test Environment I removal of redundant nodes; (**b**) Test Environment I local path smoothing; (**c**) Test Environment II removal of redundant nodes; (**d**) Test Environment II local path smoothing. The blue point represents the starting point (1, 1, 1), the yellow point represents the endpoint (100, 100, 100), blue lines show paths before optimization, red lines indicate paths after removing redundant nodes, and green lines represent the final optimized paths.

In the same test environment, a local path optimization method is introduced to further enhance the quality of the path planning results. The optimization results are illustrated in Figure 11. In Figure 11a,c, the paths before optimization are shown as blue lines, while the paths after removing redundant nodes are represented by red lines. Figure 11b,d display the final paths after local optimization, indicated by green lines.

As observed from the figure, the removal of redundant nodes leads to fewer turning points in the path, reducing its complexity. This, in turn, minimizes potential wear and tear and enhances the stability of the robotic arm during movement. The process not only improves the feasibility of the path but also effectively shortens the path length, enabling the robotic arm to reach the target point more quickly during tasks.

After local optimization, the final generated path (green line) exhibits smoother characteristics. This process specifically targets each turning point, employing smoothing algorithms like cubic B-spline curves to create more natural transitions, thereby avoiding abrupt directional changes. This optimization enhances both movement efficiency and operational accuracy, particularly in complex environments, effectively reducing the risk of collisions with obstacles. To ensure the reliability of the results, each experimental group was conducted 100 times, with the final results represented by the average values in Table 5.

Table 5 presents data related to path planning across two test environments, including the unsmoothed average path length, the unsmoothed average number of nodes, the average path length after removing redundant nodes, the average number of nodes post-redundancy removal, and the average path length following path smoothing. In Test Environment I, the unsmoothed average path length is 203.44 mm, with an average of 28.73 nodes. After removing redundant nodes, the average path length significantly decreases to 193.00 mm, and the node count reduces to 19.61, representing reductions of 5.13% and 31.73%, respectively. Although the average path length slightly increases to 199.67 mm after smoothing, the overall path structure is optimized to better accommodate the robotic arm’s motion requirements.

In Test Environment II, the unsmoothed average path length is 192.62 mm, with an average of 22.25 nodes. Similar to the first environment, removing redundant nodes reduces the average path length to 187.77 mm and the number of nodes to 15.23, reflecting reductions of 2.52% and 31.55%, respectively. Following the smoothing process, the path length slightly increases to 193.75 mm. This indicates that while the total path length grows after optimization, the process effectively reduces path curvature, enhancing both the smoothness and the stability of the robotic arm’s movement. Overall, the removal of redundant nodes and the smoothing process not only lessen the complexity and computational burden of the path but also improve its operability.

To further quantitatively analyze the impact of redundant node removal and the path smoothing process on path planning, the experiments compare the path smoothing effect both with and without redundant node removal. Key metrics, such as the number of nodes, path length, and computation time, are evaluated. The experimental data are presented in Table 6.

In terms of path length, in Test Environment I, the path length increased by approximately 0.36% when redundant nodes were not removed. However, after removing the redundant nodes, the path length decreased by 5.64%, and after smoothing, the overall path length was further shortened by 0.64%. A similar trend was observed in Test Environment II: the path length increased by 1.63% without removing redundant nodes, decreased by 2.33% after removing redundant nodes, and was further shortened by 1.50% after smoothing. These results indicate that removing redundant nodes effectively reduces path complexity and significantly optimizes path quality.

In terms of the number of path nodes, the number of nodes decreased from 28.44 to 16.96 in Test Environment I and from 20.04 to 11.54 in Test Environment II, representing reductions of 40.34% and 42.42%, respectively. This reduction improves the efficiency of path planning.

Regarding smoothing time, in Test Environment I, the smoothing time without removing redundant nodes was 9.96 × 10⁻⁴ seconds, but it decreased to 5.98 × 10⁻⁴ seconds after removing redundant nodes, a reduction of about 39.96%. In Test Environment II, the smoothing time decreased from 1.321 × 10⁻^3^ s to 6.64 × 10⁻⁴ seconds, a reduction of 49.70%. These results further demonstrate that removing redundant nodes not only optimizes the paths but also reduces the computation time of the smoothing process, thereby improving the efficiency of the overall algorithm.

### 4.4. Experiment in Tea Plantation Environment

**Figure 12:**
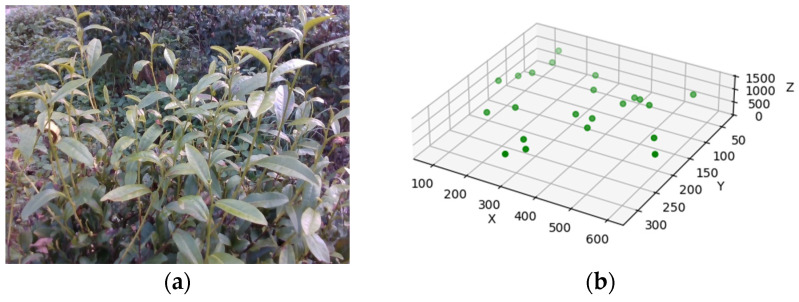
Distribution of tea buds in the picking area: (**a**) images captured by depth camera sensor; (**b**) 3D distribution of tea bud-picking points.

The tea plantation at the High-Tech Agricultural Technology Park of Anhui Agricultural University served as the experimental site. Using depth vision sensors, 200 images in JPG format with a resolution of 640 × 480 pixels were acquired for data collection on the tea plantation environment. These images captured three-dimensional information about the environment and the location coordinates of multiple tea shoot-picking points. Research indicates that tea shoots are primarily located in the upper layer of the tea trees, exhibiting a uniform horizontal distribution and minimal variation in the vertical distribution. The planting parameters of the tea garden are approximately as follows: tea ridge width is about 800 mm, and the height ranges from 800 mm to 1200 mm. The distribution of tea buds in the picking area is illustrated in Figure 12. Specifically, Figure 12a presents an image of the tea bud-picking area captured by the depth vision sensor, while Figure 12b displays the 3D coordinate distribution of the picking points within this area. Multiple testing areas were selected for the actual experiment, and Figure 12 showcases the results from only one of these areas.

**Figure 13:**
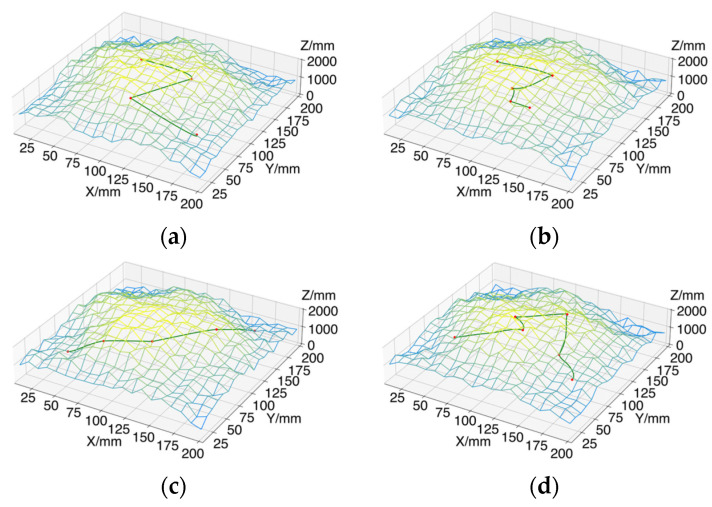
AS-RRT* path planning results in different tea plantation environments, where the number in parentheses indicates the number of picking points in each environment: (**a**) Tea Garden Environment I (4 picking points); (**b**) Tea Garden Environment II (5 picking points); (**c**) Tea Garden Environment III (5 picking points); (**d**) Tea Garden Environment IV (6 picking points). The red points represent tea-picking locations, green lines show the planned paths, and the blue grid displays the tea plantation’s 3D information.

To enhance the efficiency of path planning, an enclosing box and a center point are first generated for each picking point using the nearest-neighbor algorithm. Independent path planning is then conducted for each enclosing box. This method decomposes the environment into smaller regions, making path planning more targeted, reducing collision risks, and improving the robotic arm’s real-time responsiveness for tea picking. Additionally, this approach alleviates the computational burden while ensuring that the robotic arm effectively navigates obstacles in complex environments, thereby enhancing operational safety and flexibility. In the path search process for each enclosing box, four different tea plantation environments, each measuring 200 mm × 200 mm × 2000 mm, are established. To ensure real-time picking capabilities, the maximum number of iterations is set to 1000, and the initial step size is configured to 20 mm based on the coordinate range of the picking points. Each experimental group is repeated 100 times, and the final path planning results are derived by averaging the outcomes. In Figure 13, the red points represent the tea-picking locations, the green lines illustrate the path planning results, and the blue grid displays three-dimensional information about the tea plantation environment.

Table 7 summarizes the performance comparison between the AS-RRT* algorithm and the traditional RRT algorithm regarding path planning efficiency and effectiveness across different tea plantation environments. In Tea Plantation Environment I, which has 4 picking points, the average path length for the RRT* algorithm is 442.72 mm, whereas the AS-RRT* algorithm achieves a reduced path length of 399.88 mm, highlighting its superiority in minimizing the distance traveled by the robotic arm. Additionally, the planning time for the AS-RRT* algorithm is 0.71 s, significantly lower than the RRT* algorithm’s 8.30 s, demonstrating its enhanced real-time performance in complex environments.

In Tea Plantation Environment II, which includes 5 picking points, the average path length for the RRT* algorithm is 268.70 mm, while the AS-RRT* algorithm reduces this to 252.15 mm, further demonstrating the effectiveness of the optimized paths. The planning time for the AS-RRT* algorithm is only 0.30 s, representing a significant efficiency improvement compared to the 1.69 s required by the RRT algorithm. In Environment III, also with 5 picking points, the average path length for the RRT* algorithm is 436.41 mm, whereas the AS-RRT* algorithm optimizes it to 405.73 mm, with a planning time of 0.63 s, compared to the RRT* algorithm’s planning time of 3.52 s.

In Environment IV, which features 6 picking points, the average path length of the standard RRT algorithm measures 495.71 mm. In contrast, the AS-RRT algorithm successfully reduces this path length to 434.32 mm, achieving a reduction of 12.38%. Additionally, the planning time for the AS-RRT* algorithm is significantly shortened to 0.78 s, highlighting its effectiveness in rapidly responding to the complexities of the environment.

The Euclidean distances of the AS-RRT* algorithm exhibited less deviation across different environments, indicating that the generated paths are closer to the actual picking points. This enhancement increases both the feasibility of the paths and the accuracy of the operation. The results demonstrate that the AS-RRT* algorithm not only optimizes path generation but also minimizes unnecessary movements and adjustments during operation, thereby improving the efficiency of tea picking. In conclusion, the data presented in Table 4 not only confirm the advantages of the AS-RRT* algorithm in terms of path length and planning time but also underscores its effectiveness in achieving efficient path planning in complex tea plantation environments, providing robust support for the application of robotic arms in tea picking.

### 4.5. Tea-Picking Robotic Arm Motion Planning Experiment

**Figure 14:**
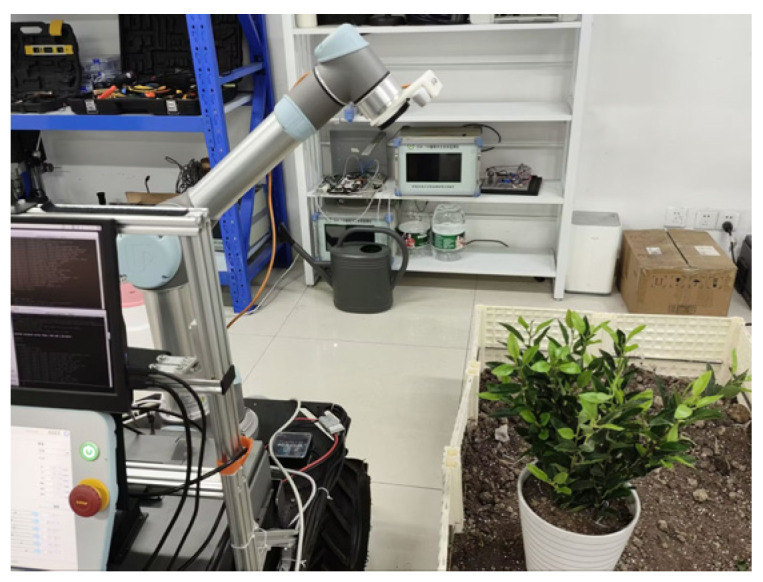
Random point sampling process.

To evaluate the performance of the AS-RRT* algorithm in complex picking scenarios, a simulated picking environment was constructed in the laboratory, as shown in Figure 8. The design of the simulated tea leaves carefully considered the requirements of actual tea-picking tasks to replicate the characteristics of picking points and the flexible structure of tea leaves. The experimental setup consisted of a UR5 robotic arm (load capacity: 5 kg, repeat positioning accuracy: ±0.03 mm, maximum arm span: 850 mm) and an Intel RealSense D435i depth vision sensor. The depth vision sensor was mounted at the end of the robotic arm to capture positional information on the target tea leaves (as depicted in Figure 14). The initial configuration of the robotic arm is set to (0°, −90°, −50°, −90°, −90°, 0°). This configuration is designed to enable the robotic arm’s end-effector to quickly reach the picking point while ensuring that the depth vision sensor at the end can effectively capture the tea plantation environment information. By positioning the arm in a diagonal downward direction, the motion flexibility is maximized, thereby ensuring an efficient picking process.

**Figure 15:**
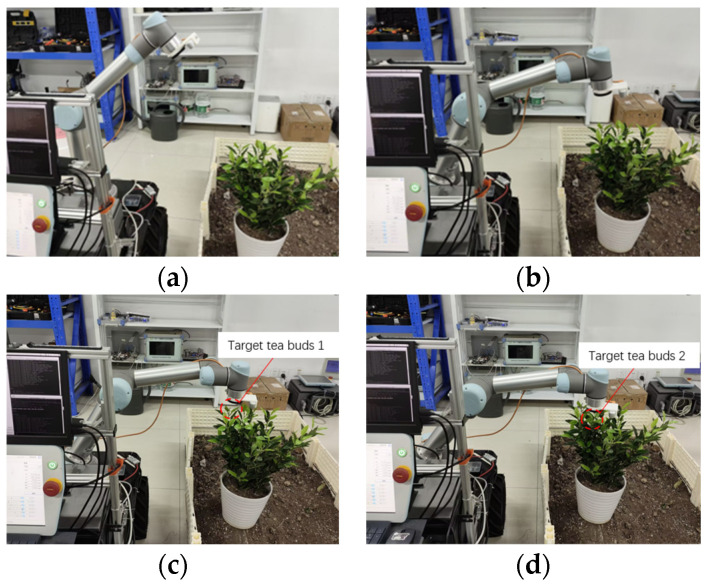
Robotic arm motion process: (**a**) initial position; (**b**) movement process; (**c**) Target Position 1; (**d**) Target Position 2.

The experiment involves creating a 3D model of the robotic arm using URDF files, which are then used to configure and initialize the trajectory planning process with the MoveIt function package. The 3D model of the robotic arm is visualized in RViz. The AS-RRT* algorithm is integrated into the open-source motion planning library, OMPL, to handle path planning and obstacle avoidance tasks. During the experiment, the robotic arm is tasked with moving from one target picking point to another. A diagram illustrating the robotic arm’s movement process is shown in Figure 15.

**Figure 16:**
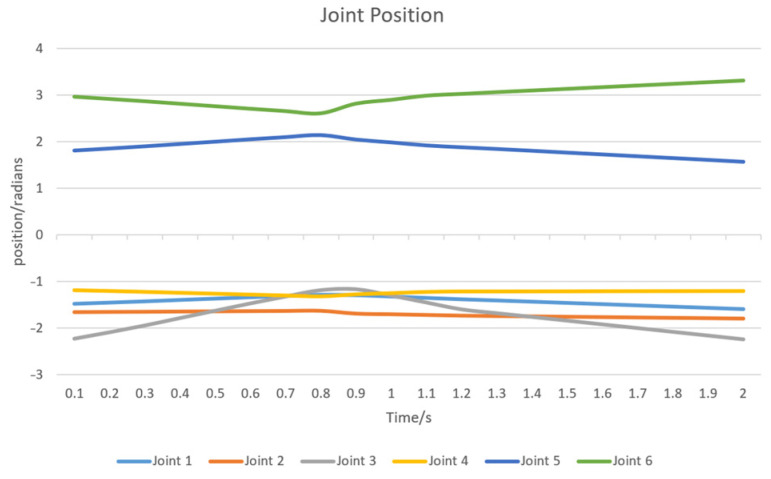
Position of each joint.

In addition, to further analyze the movement of each joint during the robotic arm’s motion, a graph depicting the changes in joint positions over time was plotted (as shown in Figure 16). The graph illustrates the trajectories of the six joints of the robotic arm throughout the path planning process. From the figure, it can be observed that joints 3, 5, and 6 exhibit significant position changes, indicating that these joints play a crucial role in the overall movement of the robotic arm. Joint 3 is primarily responsible for adjusting the mid-range posture of the arm to ensure a smooth path, while joints 5 and 6 are more involved in fine-tuning the attitude of the end-effector. The smoothness of the curves further highlights the stability and controllability of the AS-RRT* algorithm in guiding the robotic arm’s motion.

The experiment was repeated 50 times, and the statistical results include the average running time, path length, and the success rate of the robotic arm in completing the task (as shown in Table 8).

The experimental data demonstrate that the improved AS-RRT* algorithm significantly outperforms the conventional RRT* algorithm in terms of performance. The runtime is reduced by approximately 36.84% (from 1.14 s to 0.73 s), the path length is shortened by around 14.18% (from 103.66 mm to 89.03 mm), and the success rate of the robotic arm completing the task is improved by about 9.30% (from 86.00% to 95.00%). The reduction in runtime suggests that the AS-RRT* algorithm optimizes the sampling strategy and node selection efficiency during the path search process, while the decrease in path length indicates its ability to generate better trajectories, reducing the robotic arm’s motion redundancy. Furthermore, the improved success rate further confirms the robustness and reliability of the AS-RRT* algorithm in complex environments, particularly in obstacle-dense scenarios. These experimental results highlight that the AS-RRT* algorithm can effectively plan paths, avoid obstacles, and ensure the robotic arm safely reaches the target picking point.

## 5. Conclusions

This study proposes the Adaptive Step RRT* (AS-RRT*) algorithm to enhance the path planning of a tea-picking robotic arm in a tea garden. The developed sampling point selection strategy is designed and validated to optimize both the efficiency of path planning and the quality of the generated paths. By adjusting the strategy post-collision detection, the robot can safely avoid obstacles. Furthermore, the introduction of redundant node removal and local cubic B-spline curve smoothing techniques further optimizes the generated path, resulting in smoother motion of the robotic arm.

The results of the 3D algorithm simulation experiments indicate that the AS-RRT* algorithm achieves a 7.73% reduction in path cost, a 56.00% decrease in running time, and a 33.33% reduction in the number of sampling nodes compared to the RRT* algorithm. In experiments conducted in the tea plantation environment, the proposed method effectively shortens the length of the final path by 12.38% and reduces the time required for path planning. These enhancements significantly improve the path planning performance of the tea-picking robot. Through practical verification on the UR5 robotic arm, the path planned by the AS-RRT* algorithm is shown to be not only highly efficient but also effective in reducing the final path length by 14.18%. Additionally, it ensures the smoothness and reliability of the robotic arm’s movement throughout the process.

## Figures and Tables

**Table 1:** UR5 D-H parameters table.

Joint i	α_i−1_/rad	a_i−1_/m	θ_i−1_/rad	d_i_/m
Joint 1	π/2	0	0	0.089159
Joint 2	0	−0.425	0	0
Joint 3	0	−0.39225	0	0
Joint 4	π/2	0	0	0.10915
Joint 5	−π/2	0	0	0.09465
Joint 6	0	0	0	0.0823

**Table 2:** Step size mean and variance.

Environments	Step Size Mean/mm	Step Size Variance/s
Test Environment I	5.73	0.52
Test Environment II	4.92	0.498

**Table 3:** Test Environment I path planning results.

Algorithm	Path Length/mm	Number of Nodes/pc	Number of Sampling Nodes/pc	Number of Effective Sampling Nodes/pc	Planning Time/s
RRT*	206.29	29.90	328.61	215.95	0.93
AS-RRT*	193.00	19.61	278.02	159.58	0.51

**Table 4:** Test Environment II path planning results.

Algorithm	Path Length/mm	Number of Nodes/pc	Number of Sampling Nodes/pc	Number of Effective Sampling Nodes/pc	Planning Time/s
RRT*	203.50	31.17	257.07	188.39	1.25
AS-RRT*	187.77	15.23	171.37	110.71	0.55

**Table 5:** Three-dimensional environment path optimization results.

Test Environment	Original Path/mm	Original Path Nodes/pc	Path Length After Redundant Nodes Removal/mm	Nodes After Removal/pc	Smoothed Path Length/mm
Test Environment I	203.44	28.73	193.00	19.61	199.67
Test Environment II	192.62	22.25	187.77	15.23	193.75

**Table 6:** Path smoothing effect with and without removing redundant nodes.

Test Environment	Original Path/mm	Original Path Nodes/pc	Path Length After Redundant Nodes Removal/mm	Nodes After Removal/pc	Smoothed Path Length/mm	Planning Time/s	Smoothing Time/s
Env I (No Redundancy)	206.62	28.44	/	/	207.37	0.74	9.96 × 10⁻⁴
Env I (Redundancy Removed)	206.06	28	194.45	16.96	204.74	0.73	5.98 × 10⁻⁴
Env II (No Redundancy Removed)	192.91	20.04	/	/	196.04	0.72	1.321 × 10⁻^3^
Env II (Redundancy Removed)	191.70	19.74	187.22	11.54	194.82	0.71	6.64 × 10⁻⁴

**Table 7:** Results of environmental path planning for tea plantations.

Test Environment	European Distance/mm	Algorithm	Average Path Length/mm	Planning Time/s
Tea Garden Environment I (4)	378.52	RRT*	442.72	8.30
		AS-RRT*	399.88	0.71
Tea Garden Environment II (5)	250.89	RRT*	268.70	1.69
		AS-RRT*	252.15	0.30
Tea Garden Environment III (5)	396.11	RRT*	436.41	3.52
		AS-RRT*	405.73	0.63
Tea Garden Environment IV (6)	429.65	RRT*	495.71	3.31
		AS-RRT*	434.32	0.78

**Table 8:** Robotic arm motion planning results.

Algorithm	Path Cost/mm	Running Time/s	Success Rate of Robotic Arm Movement/percent
RRT*	1.14	103.66	0.86
AS-RRT*	0.73	89.03	0.95

## Data Availability

The data in this study are available upon request from the corresponding author.
